# Membranous nephropathy in primary antiphospholipid syndrome

**DOI:** 10.1093/ckj/sfae017

**Published:** 2024-02-02

**Authors:** Romain Stammler, Camille Rapoport, Jean Paul Duong Van Huyen, Stéphane Zuily, Moranne Olivier, Eric Daugas, Emmanuel Esteve, Mathilde de Menthon, Helene Perrochia, Charlotte Mussini, Aurélie Sannier, Marion Rabant, David Buob, Alexandre Karras

**Affiliations:** AP-HP, Georges Pompidou European Hospital, Nephrology Department, Paris, France; AP-HP, Necker Hospital, Pathology Department, Paris, France; AP-HP, Necker Hospital, Pathology Department, Paris, France; Nancy University Hospital, Vascular Medicine Department, French Reference Center for Rare Systemic Auto-Immune and Auto-Inflammatory Diseases, Nancy, France; University Hospital Center of Nîmes, Nephrology Department, Nîmes, France; AP-HP, Bichat Hospital, Nephrology Department, Paris, France; AP-HP, Tenon Hospital, Nephrology Department, Paris, France; AP-HP, Bicêtre Hospital, Internal Medicine Department, Paris, France; Montpellier University Hospital, Pathology Department, Montpelier, France; AP-HP, Bicêtre Hospital, Pathology Department, Paris, France; AP-HP, Bichat Hospital, Pathology Department, Paris, France; AP-HP, Necker Hospital, Pathology Department, Paris, France; AP-HP, Tenon Hospital, Pathology Department, Paris, France; AP-HP, Georges Pompidou European Hospital, Nephrology Department, Paris, France

To the Editor,

Antiphospholipid syndrome (APS) is a systemic autoimmune disorder characterized by thrombotic and obstetrical complications associated with antiphospholipid antibodies. The spectrum of renal involvement in APS comprises several types of nephropathy, mainly vascular nephropathies [1]. Glomerular involvement has mainly been described in secondary APS associated with systemic lupus erythematosus (SLE). Nevertheless, rare cases of membranous nephropathy (MN) with APS and without any clinical or immunological characteristics of SLE have previously been reported in the literature [2, 3].

Here we report eight patients who presented with a biopsy-proven MN associated with a primary APS (Supplementary data, Table S1). The median age at nephropathy presentation was 32 (range 23–64) years. Five (62.5%) patients were female. A median delay of 40.5 (range 3–91) months was observed between APS diagnosis and MN diagnosis. A triple positivity for the three antiphospholipid antibodies tests was found in six cases (75%). Five (62.5%) and two (25%) patients, respectively, had thrombotic or obstetrical APS-related symptoms. At nephropathy diagnosis, patients displayed a mean urine protein-to-creatinine ratio of 5.6 g/g, with microhematuria in five cases (62.5%), and a mean albuminemia of 33 g/L. High blood pressure was present in five cases (62.5%), while the mean estimated glomerular filtration rate at presentation was 73.6 mL/min/1.73 m^2^. A single patient (12.5%) had circulating anti-phospholipase A2 receptor (anti-PLA2R) antibodies. No patient displayed extra-renal clinical features of SLE. Five (62%) patients had antinuclear antibodies at a titer ≥1:80 but only one patient had transient positivity of DNA antibodies, without complement consumption. No extractable nuclear antigen antibody was found. With a median follow-up of 73 months, only one patient (12.5%) developed end-stage renal disease, leading to pre-emptive kidney transplantation. None required dialysis and no death was observed. All patients demonstrated typical MN at different stages on kidney biopsy (Fig. 1). Routine immunofluorescence study found typical granular immunoglobulin G (IgG) and C3 deposits along the glomerular basement membrane in 8 (100%) and 7 (87.5%) patients, respectively. Two patients (25%) also had a few C1q deposits. Two patients had glomerular PLA2R-positive deposits, only one of whom had also circulating anti-PLA2R antibodies. Immunohistochemistry staining for thrombospondin type 1 domain-containing 7A (THSD7A), neural epidermal growth factor-like 1 (NELL-1) and exostosin was negative in all patients. Four patients (80%, missing data = 3) presented predominant glomerular deposits of IgG1 while a single patient (20%, missing data = 3) displayed IgG4 glomerular deposits. Three patients (37.5%) had histological features of classical APS nephropathy in addition to those of MN.

**Figure 1:**
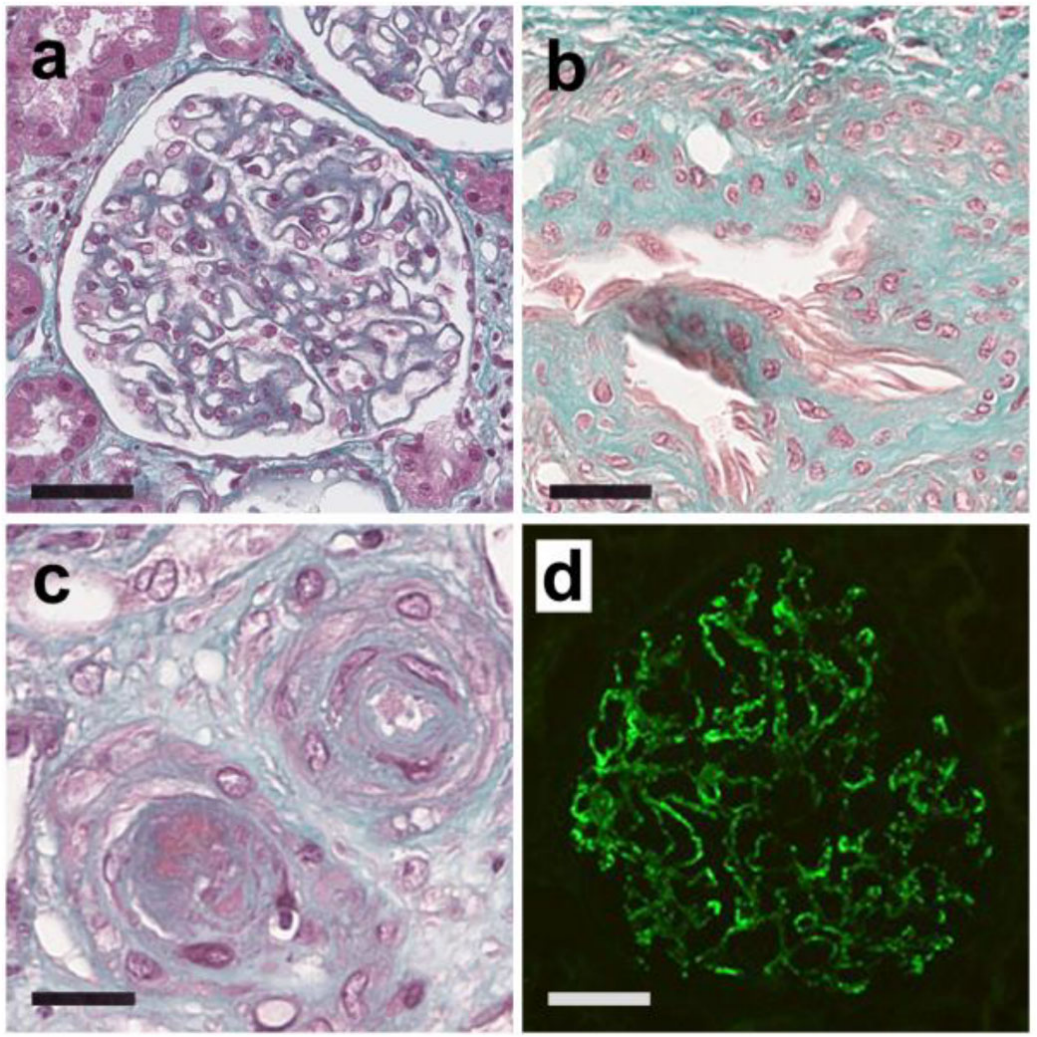
Histopathological representative images of membranous nephropathy associated to primary antiphospholipid syndrome.

Primary MN is defined by the existence of a MN without any underlying disease or associated condition [4]. Anti-PLA2R antibodies are the most common antibodies retrieved in primary MN [4]. Studies of immunoglobulins subclass in glomerular deposits usually reveals predominance of IgG4 [4]. However, about 30% of MN cases are associated with other diseases [4]. The most common autoimmune condition associated with secondary MN is SLE. Association of MN with lupus-associated APS is frequent, but data regarding occurrence of MN during primary APS are scarce to date. In the present cases, an SLE diagnosis was not retained based on the usual clinical and immunological criteria. In contrast to the usual findings in SLE-associated MN, two patients showed positive PLA2R glomerular staining on kidney biopsy, whereas none of the patients had positive exostosin staining. In addition, IgG subclass analysis revealed exclusive IgG1 deposits in 4/5 cases, whereas primary MN is usually associated with isolated IgG4 deposits and SLE-associated MN is mostly associated with multiple IgG subclass deposits [5]. Immunostaining for THSD7A and NELL-1 were also negative in all cases, possibly suggesting the presence a specific antigen in APS-associated MN that remains to be identified.

In conclusion, our results identify the co-occurrence of APS and MN which is suggestive of a potential association that remains to be confirmed.

## Supplementary Material

sfae017_Supplemental_File

## References

[1] Barbhaiya M , Taghavi M, Zuily S et al. Efforts to better characterize “antiphospholipid antibody nephropathy” for the 2023 ACR/EULAR Antiphospholipid Syndrome Classification criteria: Renal Pathology Subcommittee report. J Rheumatol 2024;**51**:150–9. 10.3899/jrheum.2022-1200. 37399462

[2] Fakhouri F , Noël L-H, Zuber J et al. The expanding spectrum of renal diseases associated with antiphospholipid syndrome. Am J Kidney Dis 2003;41:1205–11. 10.1016/S0272-6386(03)00352-4. 12776272

[3] Sinico RA , Cavazzana I, Nuzzo M et al. Renal involvement in primary antiphospholipid syndrome: retrospective analysis of 160 patients. Clin J Am Soc Nephrol 2010;5:1211–7. 10.2215/CJN.00460110. 20430943 PMC2893064

[4] Sethi S , Beck LH, Glassock RJ et al. Mayo Clinic consensus report on membranous nephropathy: proposal for a novel classification. Kidney Int 2023;104:1092–102. 10.1016/j.kint.2023.06.032. 37795587

[5] Song YS , Min K-W, Kim JH et al. Differential diagnosis of lupus and primary membranous nephropathies by IgG subclass analysis. Clin J Am Soc Nephrol 2012;7:1947–55. 10.2215/CJN.04800511. 23024158 PMC3513749

