# Supplementary material for: Membranous nephropathy in primary antiphospholipid syndrome
Source: Clin Kidney J. 2024 Feb 2;17(2):sfae017. doi: 10.1093/ckj/sfae017 (PMC10872675; doi:10.1093/ckj/sfae017)
Supplement: sfae017_Supplemental_File [file sfae017_supplemental_file.docx]

| **Case number** | **#1** | **#2** | **#3** | **#4** | **#5** | **#6** | **#7** | **#8** |
| --- | --- | --- | --- | --- | --- | --- | --- | --- |
| **Clinical and biological characteristics** | | | | | | | | |
| **Sex** | M | F | F | M | M | F | F | F |
| **Age at diagnosis of nephropathy (years)** | 30 | 64 | 23 | 34 | 59 | 30 | 40 | 27 |
| **APS extrarenal clinical criteria** | - | DVT/PE | HELLP syndrome leading to late miscarriage | Multiple strokes | PE | Venous and arterial thrombosis | Obstetrical complications | Obstetrical complications |
| **APS preceding nephropathy** | - | - | - | - | APS 23 months before MN | NA | NA | APS 13 months before MN |
| **Delay between APS classification and nephropathy (months)** | 91 | 3 | 52 | 29 | 23 | NA | NA | 13 |
| **Proteinuria (g/g of urine creatinine)** | 16 | 4.7 | 1.3 | 14.0 | 4.7 | 1.35 | 0.3 | 2.5 |
| **Hematuria** | + | + | - | - | + | - | + | + |
| **eGFR (mL/min/1.73m2)** | 18 | 26 | 90 | 112 | 43 | 90 | NA | 136 |
| **Albuminemia (g/L)** | 28 | 41 | 40 | NA | 26 | NA | NA | 28 |
| **Blood pressure (systolic/diastolic)** | 220/130 | 171/69 | 120/64 | 160/105 | 135/80 | 141/98 | 162/110 | 110/54 |
| **Persistent LA** | + | + | + | + | + | + | + | + |
| **ACL (moderate or high titer)** | + | + | + | + | - | + | + | + |
| **Anti-B2GP1 (moderate or high titer)** | + | + | + | - | - | + | + | + |
| **ANA** | 1:320 | 1:640 | 1:160 | 1:120 | 0 | 0 | 0 | 1:160 |
| **Anti-DNA** | - | + | - | - | - | - | - | - |
| **Anti-PLA2R** | - | - | - | - | + | - | - | - |
| **Outcome** |  |  |  |  |  |  |  |  |
| **Dialysis** | - | - | - | - | - | - | - | - |
| **Kidney transplantation** | + | - | - | - | - | - | - | - |
| **Death** | - | - | - | - | - | - | - | - |
| **Follow up (months)** | 100 | 44 | 124 | 120 | 46 | 220 | 19 | 24 |
| **Pathology** |  |  |  |  |  |  |  |  |
| **Membranous nephropathy** | Stage 3 MN | Stage 2 MN | Stage 3 MN | Stage 2 MN | Stage 2 MN | Stage 2 MN | Stage 2 MN | Stage 1 MN |
| **APSN associated features** | Arteriolar TMA | 0 | 0 | 0 | 0 | Arteriolar TMA, Intimal fibrocellular hyperplasia | Intimal fibrocellular hyperplasia | 0 |
| **MN specific treatment** | - | Rituximab | - | Steroids, cyclophosphamide | Rituximab | Steroids | Steroids, mycophenolate mofetil, hydroxychloroquine | - |
| **IgA** | 0 | 0 | 0 | 0 | 0 | 0 | 0 | 0 |
| **IgG** | + | + | +++ | + | ++ | ++ | +++ | +++ |
| **IgM** | 0 | 0 | + | 0 | 0 | + | 0 | 0 |
| **C3** | + | + | +++ | + | ++ | 0 | +++ | + |
| **C1q** | + | 0 | + | 0 | 0 | 0 | 0 | 0 |
| **kappa** | + | + | + | + | + | ++ | + | + |
| **lambda** | + | + | + | + | + | + | + | + |
| **IgG1 / IgG2 / IgG3 / IgG4** | + / 0 / 0 / 0 | 0 / 0 / 0 / + | ++ / 0 / + / 0 | NA | 0 / 0 / 0 / + | + / 0 / 0 / 0 | + / 0 / 0 / 0 | + / 0 / 0 / 0 |
| **PLA2R** | 0 | 0 | 0 | + | + | 0 | 0 | 0 |
| **THSD7A** | 0 | 0 | 0 | 0 | 0 | 0 | 0 | 0 |
| **EXT** | 0 | 0 | 0 | 0 | 0 | 0 | 0 | 0 |
| **NELL1** | 0 | 0 | 0 | 0 | 0 | 0 | 0 | NA |

**Supplemental Table 1: Clinical and histopathological characteristics of the 8 patients with membranous nephropathy associated to primary antiphospholipid syndrome**

Legends: ACL: anticardiolipin; ANA: antinuclear antibody; APS: antiphospholipid syndrome; B2GP1: beta-2 glycoprotein-1: DNA: deoxyribonucleic acid; DVT: deep venous thrombosis; eGFR: estimated glomerular filtration rate; EXT: exostosin; F: female; HELLP; hemolysis elevated liver enzymes low platelet count; Ig: immunoglobulin; LA: lupus anticoagulant; M: male; MN: membranous nephropathy; NA: not available; NELL1: neural EGFL like-1; PE: pulmonary embolism; PLA2R: phospholipase A2 receptor; THSD7A: thrombospondin 7A; TMA: thrombotic microangiopathy
